# Development of a Patient-Specific Finite Element Model for Predicting Implant Failure in Pelvic Ring Fracture Fixation

**DOI:** 10.1155/2017/9403821

**Published:** 2017-02-01

**Authors:** Vickie Shim, Andreas Höch, Ronny Grunert, Steffen Peldschus, Jörg Böhme

**Affiliations:** ^1^Auckland Bioengineering Institute, University of Auckland, 70 Symonds Street, Auckland, New Zealand; ^2^Menzies Health Institute, Griffith University, Gold Coast, QLD, Australia; ^3^Department of Trauma, Plastic and Reconstructive Surgery, University of Leipzig, Liebigstr. 20, 04103 Leipzig, Germany; ^4^Institute of Forensic Medicine, Ludwig-Maximilians-University Munich, Munich, Germany

## Abstract

*Introduction.* The main purpose of this study is to develop an efficient technique for generating FE models of pelvic ring fractures that is capable of predicting possible failure regions of osteosynthesis with acceptable accuracy.* Methods.* Patient-specific FE models of two patients with osteoporotic pelvic fractures were generated. A validated FE model of an uninjured pelvis from our previous study was used as a master model. Then, fracture morphologies and implant positions defined by a trauma surgeon in the preoperative CT were manually introduced as 3D splines to the master model. Four loading cases were used as boundary conditions. Regions of high stresses in the models were compared with actual locations of implant breakages and loosening identified from follow-up X-rays.* Results.* Model predictions and the actual clinical outcomes matched well. For Patient A, zones of increased tension and maximum stress coincided well with the actual locations of implant loosening. For Patient B, the model predicted accurately the loosening of the implant in the anterior region.* Conclusion.* Since a significant reduction in time and labour was achieved in our mesh generation technique, it can be considered as a viable option to be implemented as a part of the clinical routine to aid presurgical planning and postsurgical management of pelvic ring fracture patients.

## 1. Introduction

Osteoporotic pelvic fractures are increasing rapidly in developed countries. An international survey forecasts that the overall incidence of osteoporotic pelvic fracture will increase rapidly, with women over 85 years of age being at the highest risk [1]. Gänsslen [2] reported that patients with osteoporotic pelvic ring fractures can be treated with the same surgical procedures as nonosteoporotic patients. They drew their conclusions from the biomechanical testing of osteosynthesis performed on cadaveric pelvises from donors with average age of 70 years old, which the authors considered to be indicative of osteoporosis [2]. Although tests on cadaveric pelvises are still the gold standard in orthopaedic biomechanics testing, low availability of donor pool, coupled with high interindividual variations in the geometry and material properties, limits the validity of applying the results to cases other than those actually tested [3, 4]. And this problem is exacerbated for the case of osteoporotic bones, which are extremely difficult to handle due to their brittle nature. The finite element method (FEM), on the other hand, can be of great benefit in overcoming these problems as it can provide uniform testing standards for investigating the influence of various different geometric or material parameters. However, finite element (FE) simulations of the stability of osteosynthesis at the pelvic ring have been investigated by only a few groups so far [5–8].

Previously, we demonstrated that patient-specific FE models could be used to predict the failure of osteosynthesis in surgically stabilized osteoporotic pelvic fractures by comparing model prediction with actual clinical cases [9]. However, due to the highly complicated fracture patterns, the FE model in that study was created entirely by manual processes, which made the actual clinical use of the model unrealistic. Therefore, the main purpose of this study is to develop an efficient technique for generating FE models of patients with pelvic ring fractures. We aim to achieve the following two objectives: (1) the model should be able to make qualitative predictions of possible failure regions of osteosynthesis and (2) the model needs to reduce the simulation time to the level compatible to be used in clinical environment without sacrificing the validity of our previous models.

## 2. Patients and Methods

### 2.1. Study Type, Course, and Data

A prospective, nonrandomized single center observational study was done in Level 1 trauma center of a German university hospital. The inclusion criteria were those with pelvic ring fracture without need for reduction who are over 65 years of age and female with pain killer resistance. Written consent was obtained from the patients. Exclusion criteria included polytrauma (injury severity score >18) and incompliance. The recruitment period was four months and the follow-up period 12 months. The following data were recorded as follows:

*Documented Study Data*

- Preoperative data:
  - Accident mechanism
  - Age, sex, body size, and weight
  - Fracture morphology (CT)
- Intraoperative data:
  - Implant material
- Postoperative data (2 days after surgery):
  - Implant position (CT)
- Data of the numerical simulation:
  - Identification of local stress zones in the bone or implant
- Data of follow-up (8 weeks and 3, 6, and 12 months postoperatively):
- Failure of osteosynthesis with implant breakage or dislocation (plane X-ray)

After checking the inclusion and exclusion criteria, the surgical stabilization followed. Here, the geometric and material parameters of the implants were obtained from the manufacturer's specification. Based on the pre- and postoperative CT scans of the pelvis with a 0.5 mm gap between slices (Brilliance, Royal Philips Electronics, Amsterdam, Netherlands), the pre- and postoperative fracture line and the implant position were recorded. Then, after the postoperative CT scanning, the fracture lines were implemented and implants were inserted according to the patient CT scans on the existing FE master model (see Section 2.3.1 for detailed description). Then FE analysis was done in parallel with the actual clinical course to compare FE predictions with the clinical outcomes. This study was approved by the ethics committee of the University of Leipzig (392-11-12122011).

### 2.2. Patients

**Figure 1:**
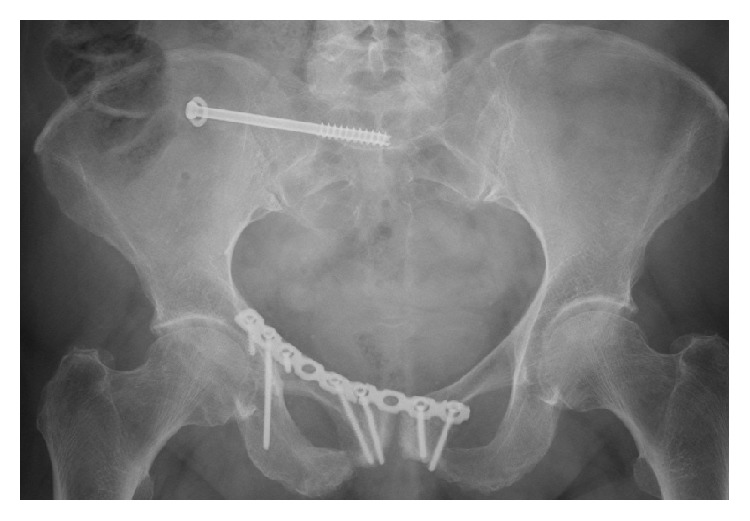
Postoperative X-ray 6 months after surgery.

*Patient A.* The 70-year-old woman suffered a low-energy trauma and then a lateral compression fracture of the pelvis (AO-61 B2.1.1-fracture) with a transpubic and transsacral instability right. Because of painkiller-resistant complaints, the Open Reduction and Internal Fixation (ORIF) was performed using 3.5 mm 9-hole titanium reconstruction plate (DePuy Synthes Comp., Zuchwil, Switzerland) via modified Stoppa access in the anterior pelvic ring and a 3D computer-navigated transiliosacral screw in S1 using 7.3 mm titanium screw with 32 mm thread length (DePuy Synthes Comp., Zuchwil, Switzerland) in the posterior pelvic ring. Full weight bearing was allowed postoperatively. During the regular follow-up, eight weeks postoperatively, loosening of the screws in the anterior pelvic ring (Figure 1) was detected. Dislocation of the fracture was not detected. Six months after surgery a plate breakage in the anterior pelvic ring (Figure 1) was evident. In the follow-up, the patient did not suffer any other falls. During the further course, there were no further complications.

**Figure 2:**
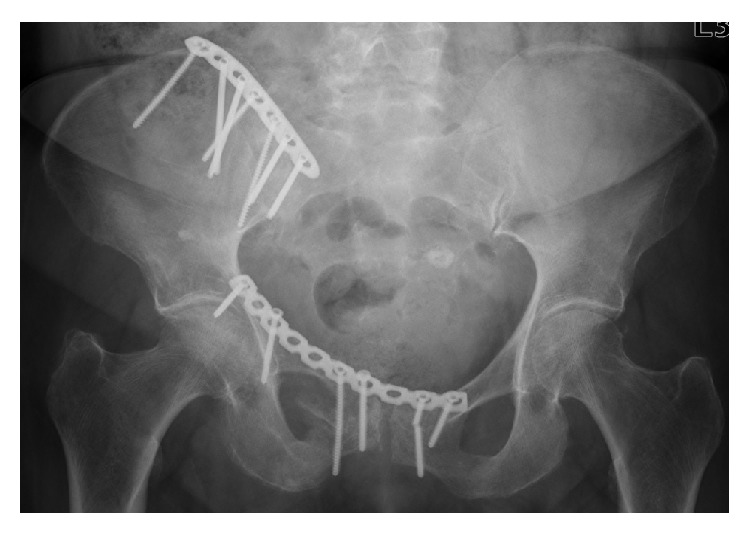
Postoperative X-ray 3 months after surgery.

*Patient B.* The 86-year-old woman suffered a collision with a car as a pedestrian (walking speed), a vertically unstable pelvic fracture (AO 61 C1.2.3) with transiliosacral and transpubic instability right. Due to the unstable fracture situation, the ORIF took place in the anterior pelvic ring with a 3.5 mm 10-hole titanium reconstruction plate (DePuy Synthes Comp., Zuchwil, Switzerland) and in the posterior pelvic ring with a 3.5 mm 7-hole titanium LCDC locking plate (DePuy Synthes Comp., Zuchwil, Switzerland). Full weight bearing was allowed postoperatively. The regular follow-up of 3 months after surgery showed both multiple loosening and the breakage of a screw in the anterior pelvic ring (Figure 2). Also no more fall events occurred during the course and neither did further complications.

### 2.3. FE Model Creation

#### 2.3.1. Creation of a Master Model

The master model was generated based on a previously developed and validated method [8–10]. First, using a CT data set of an uninjured pelvis of a 72-year-old patient in 0.5 mm table feed (Brilliance, Royal Philips Electronics, Amsterdam, Netherlands), geometric models of the hip bones and the sacrum were created semiautomatically in STL format (MIMICS, Materialise Comp., Leuven, BE) and then converted to solids in STEP format (CATIA V5, Dassault Systemes, Véllizy-Villacoublay, FR). By prior scaling of the STL meshes volume bodies of varying sizes for cortical and cancellous bone were achieved. Using Boolean operations, the cancellous bone was subtracted from the cortex, thereby obtaining separate bodies. The sacroiliac joints and the symphysis were created as an extrusion body (ANSYS Workbench 14, ANSYS Inc., Canonsburg PA, USA) and adjusted via Boolean operations on the bone contours. The sacroiliac joint was modeled with the contact type “bonded.” This compound did not allow relative motion or some other nonphysiological movement. This is a linear contact, which takes a comparatively small computational time. The contribution of the ligaments of the pelvis was implemented according to the values reported in our previous works [11]. The following ligaments were fitted (Table 1). The ligament apparatus was inserted in the master model with 62 groove joints. Each groove joint is provided with the parameters of the ligament over one APDL-script. The groove joints were each placed between “remote points,” which are defined along the edges of the model, so that an anatomical correlation is given. Thereby, also the direction of the force of the groove joint is defined. For the material properties of cortical and cancellous bone as well as cartilage, representative average values were used shown in Table 2 [12–15].

The material properties of the ligaments were obtained from our previous studies [9]. The meshing of the bone and cartilage was performed using surface-dependent method via tetrahedral volume elements (element type Solid 186) with a central node for both cortical and for cancellous bone, the ligaments with element type link 180. The total number of nodes in a mesh was 189,883 nodes. A consistent holding of the promontory of the sacrum was assumed as a part of the fixed boundary conditions.

#### 2.3.2. Creating a Patient-Specific Fracture Model

The novel aspect of the current study is that we used the existing master model (described in Section 2.3.1) and used fracture morphology and implant positions from preoperative CT scans to incorporate patient-specificity to the mater model as described below.

*Fracture Morphology and Implant Position.* The layered fracture line defined by a trauma surgeon in the preoperative CT was manually introduced as 3D splines (CATIA) to the master model of uninjured pelvis and extruded through the bone. In these divisions, the bony components were separated and saved as individual parts in STEP format. Then, the uninjured regions of the master model were replaced with the newly defined fracture regions (ANSYS Workbench 14), giving the bony geometry of a fracture model. The geometrical dimensions of the implants were taken from the manufacturer's specification. In CATIA, the plates were modeled using rectangular free-form surfaces, and screws have been simplified as a cylinder without thread. The implant position was then defined by a trauma surgeon from postoperative CT images and incorporated into the fracture model.

*FE Analysis and Boundary Conditions.* ANSYS Workbench 14 (ANSYS Inc., Canonsburg PA, USA) was used. The meshing of the fracture models was carried out as the master model using surface-dependent method and tetrahedral elements with the special feature that a refinement of the element size of about 30 mm (Patient A) or 40 mm (Patient B) to 2 mm (Patient A) or 1 mm (Patient B) was made around the fracture areas. The final mesh had 277,453 nodes for Patient A and 2,172,636 nodes for Patient B. The contact between fracture fragments along the fracture lines was modeled as frictionless contact, which enables relative movements between each other and a lifting from one another but prevents penetration of the contact partners. Although no locking implants were used, the connection between the screw and fixed plates was defined as a composite contact. The same procedure was carried out in the definition of the bone-screw and plate-bone contact.

#### 2.3.3. Load Cases

The following four load cases were simulated:

- Getting up from a chair without support
- Fast walking without support
- Stair climbing without a handrail
- Stumbling

**Figure 3:**
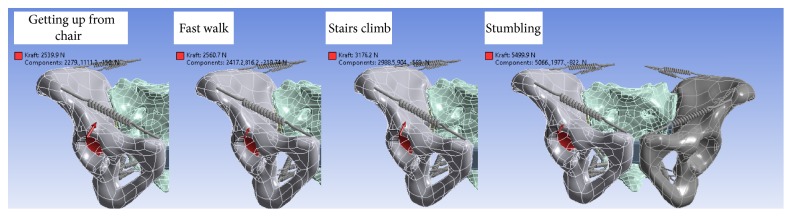
Four load cases simulated in this study.

**Figure 4:**
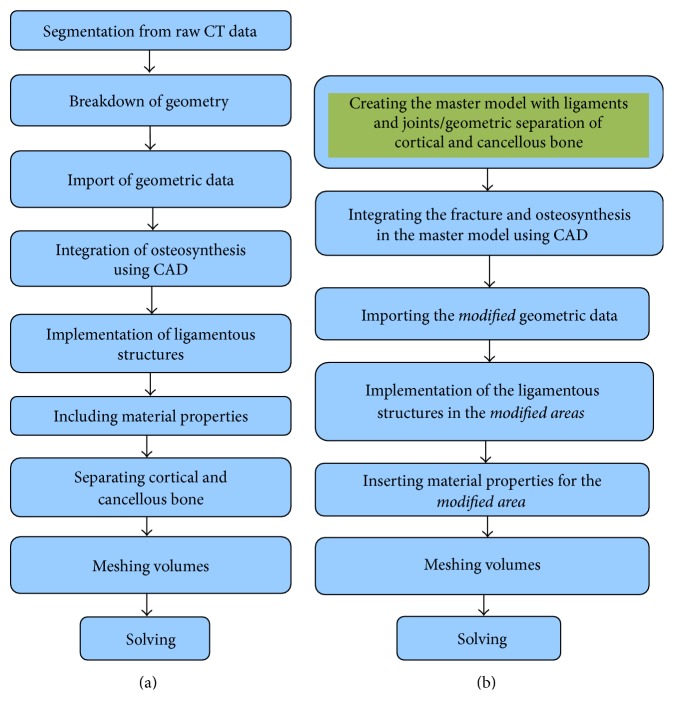
Comparison of methods for FE model creation for completely patient-specific analysis (a) and for efficient patient-specific analysis (b).

The corresponding values were obtained from the database given in Bergmann et al. [17] and converted to match the weights of the patients in this study. The force was unilaterally applied to one acetabulum except for the load case, rising without support, where the loads were applied to both sides of the acetabulum. The load cases were calculated at both the master model and the patient-specific fracture models (Figure 3). The process of generating patient-specific model is summarized in Figure 4. 

*Measured Values.* Clinically, the failure of the osteosynthesis has been defined as a fragment dislocation. Fragment dislocation refers to the displacement of fragment away from its original position achieved at the initial osteosynthesis. An implant breakage and implant loosening were identified based on the follow-up examination performed planar X-rays. Measuring implant loosening was done by a trauma surgeon (JB) as a part of their routine clinical practice. In our computational simulation, implant loosening was defined as fracture caused by permanent strain, which was determined to be strains larger than 0.3% according to the definition given by Frost [16]. In the numerical simulations, local stress zones (von Mises) and shifts were calculated with the master model and the fracture models and compared with the clinical results.

## 3. Results

### 3.1. Patients

**Figure 5:**
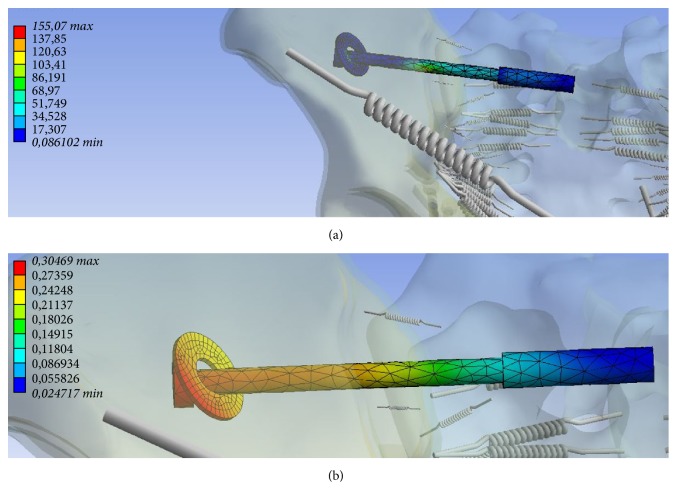
Tension ((a) in MPa, minimum 0.086 MPa, maximum 155.07 MPa) and deformation ((b) mm, minimum 0.0247 mm, maximum 0.305 mm) along the transiliosacral screw at the posterior pelvic ring.

**Figure 6:**
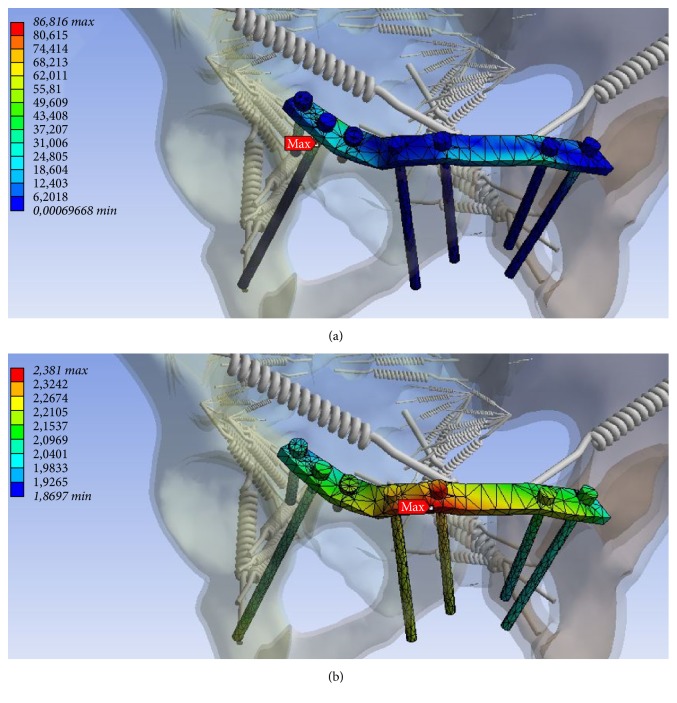
Zone of maximum tension at the infra-acetabular screw ((a) in MPa, minimum 0.0 MPa, maximum 86.816 MPa) and deformation ((b) mm, minimum 1.87 mm maximum 2.381 mm) at the plate near the right symphysis at the anterior pelvic ring.

**Figure 7:**
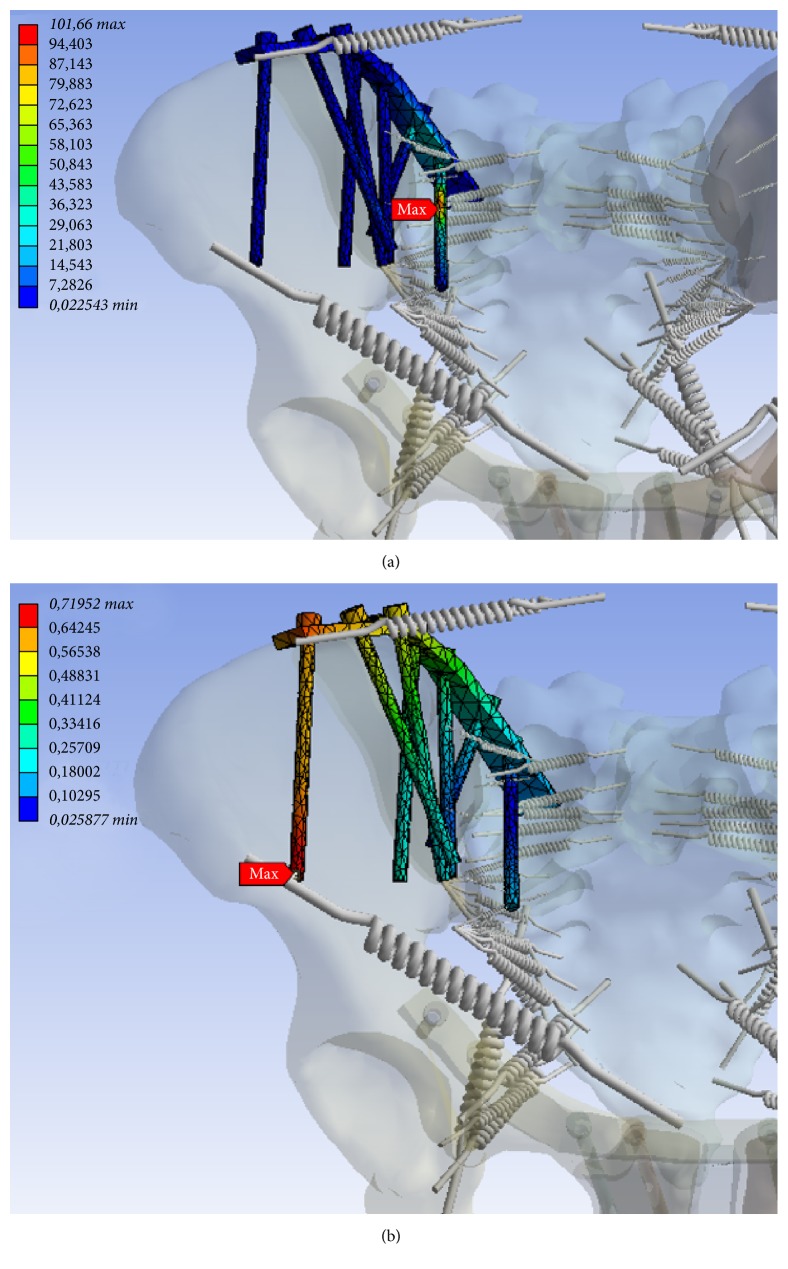
Zones of maximum tension ((a) MPa, minimum 0.0225 MPa, maximum 101.66 MPa) and deformation ((b) mm, minimum 0.0259 mm and maximum 0.71952 mm) at the osteosynthesis of the posterior pelvic ring.

**Figure 8:**
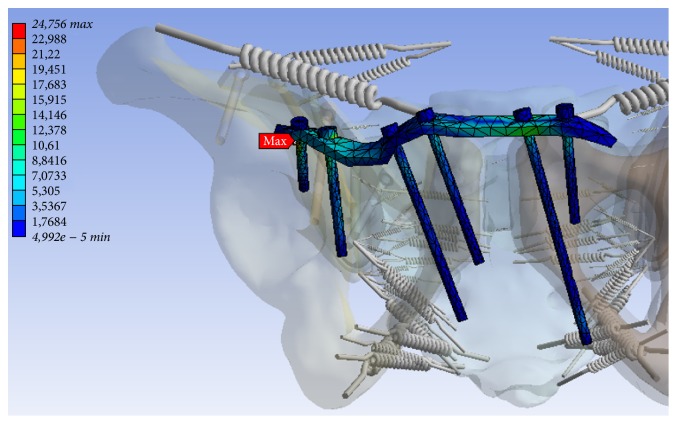
Zone of maximum tension (MPa, minimum 0.0 MPa, maximum 24.756 MPa) at the osteosynthesis of the anterior pelvic ring for Patient B.

**Figure 9:**
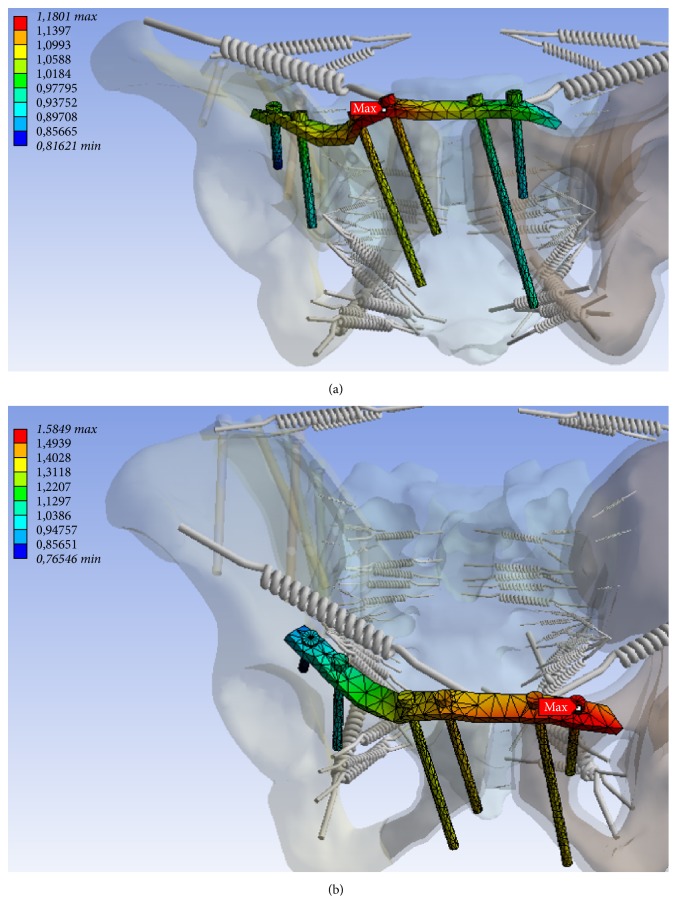
Deformation (mm) at the plate near the right (minimum 0.816 mm, maximum 1.18 mm) (a) and left symphysis (b) at the anterior pelvic ring for Patient B (minimum 0.765 mm, maximum 1.585 mm).

In the following, the results of the actual postoperative course and corresponding numerical simulations are shown. There were four simulated load cases during this study. Representative values are the results of the load case “climb stairs” and “getting up without support” which are shown in Figures 5–9.

#### 3.1.1. Patient A

Six months postoperatively, loosening of the transiliosacral screw was found in the posterior pelvic ring. In addition, there was loosening of an infra-acetabular screw and a plate breakage near the right symphysis in the anterior pelvic ring (Figure 1).

In the numerical simulation, maximum tension and maximum deformation at the transiliosacral screw in the posterior pelvic ring was detected (Table 3). The maximum values have been identified in the region where the screw passes through the sacroiliac joint (Figures 5(a) and 5(b)).

At the anterior pelvic also zones of maximum stress and deformation were determined (Table 3). Here, maximum tension at the infra-acetabular screw and maximum deformation at the plate near the right symphysis were identified (Figures 6(a) and 6(b)).

#### 3.1.2. Patient B

Three months postoperatively, loosening of the infra-acetabular screw and the screw nearby the symphysis on the right and a screw breakage near the symphysis on the left were diagnosed. Furthermore, loosening of the screw at the left superior pubic ramus was evident. At the osteosynthesis of the posterior pelvic ring, no complication was noted (Figures 6(a) and 6(b)).

In the numerical simulation, maximum stress was found to be along the medial screw (Figure 7(a)), and maximum displacement was evident at the lateral screw in the posterior pelvic ring (Figure 7(b)). In the anterior pelvic ring, maximum tension was determined at the right supra-acetabular screw (Figure 8). Also a large deformation along the plate in the near of the right and left superior pubic ramus has been identified (Figures 9(a) and 9(b)). The maximum tensions and displacements are shown in Table 4.

## 4. Discussion

Through our previous works [8–10], we have shown that numerical simulations with realistic finite element models of complex pelvic ring osteosynthesis have a potential to be able to predict the stability of osteosynthesis. Particularly in Böhme et al. [9], we demonstrated the correlation of clinical follow-up and numerical simulation in three patients by showing that zones of higher stresses lead to implant failure or breakage in pelvic ring fracture osteosynthesis [9]. In that study, a completely new and patient-specific finite element model was generated and full numerical analysis was performed. Although the results were very accurate, the whole process was very labour-intensive and time-consuming. In the current study, we significantly reduced the time and effort required in building the model so that implementing FE method into the clinical routine can be considered as a viable option. (Figure 4). Specifically, rather than generating a new model from scratch, we modified an existing finite element master model by including only the fracture zone and implant geometry and material properties using manufacturer's specification. Our approach offers advantages in the following two areas:

- Less time in model generation and solving required
- Higher resolution of moderate dislocated/displaced fractures via CAD

In this study, we focused on osteoporotic fracture because it is one of the most frequently observed fracture types in pelvic ring fractures. As such, it is the osteoporotic fractures that urgently need a novel clinical tool for better pre- and postpatient management. However, our method can be used in any type of fractures as long as the master model and preoperative CT images are available.

One major limitation of our approach is that this technique cannot be applied to severely dislocated displaced fractures because of the incomplete geometry. In such extreme cases, we do not advise to use this simplified approach. However, when dealing with pelvic ring fractures commonly seen in clinical practices, our approach potential will be of great use. In fact, in Patient A, the clinical course correlated very well with the results of the numerical simulation. Comparing the postoperative course (Figure 2) in Patient B with the results of numerical analysis, some regions showed a good match, while others showed less clear correlation. At the anterior pelvic ring zones of increased displacement in the numerical simulation correlated well with the actual implant loosening and breakage in the clinical course. However, for the posterior fixation, no correlation was evident (Figures 7(a) and 7(b)). Despite the increased tension values on the medial screw and higher degrees of displacement at the lateral screw, no loosening or failure of the internal fixation was diagnosed by X-ray. However, we suspect that the X-rays may not have enough resolution to detect the initiation of implant loosening. Also the maximum tension values predicted for Patient B ranged between 83 and 111 MPa; the maximum displacement ranged between 0,45 and 0,90 mm (Table 4) and these may not have been large enough to influence the stability of the implant-bone composite at this point.

The major strength of the technique introduced in this study is its efficiency. In our previous work [9], we used completely patient-specific finite element model and demonstrated that the zones of highest stress in the FE model matched well with the actual high stress zones in clinical cases. However, generating patient-specific finite element models of pelvic ring fracture is no trivial task. In fact, the amount of time and resources required in generating patient-specific FE model of the pelvic ring fracture and running the FE analysis makes it almost impossible to be used in clinical settings. For example, in our previous study, creating patient-specific FE model of the pelvic ring fracture and the subsequent osteosynthesis was done completely manually and it took three days by an expert user. We have improved this by using the existing master model and just importing fracture morphology and implant positions. This has dramatically improved the whole procedure by saving times in image processing, segmentation, and so forth. In fact, the new procedure only took less than two hours. Combining the running time for ANSYS FE analysis, which is about 8 hours for a standard PC, the total amount of time required for this new procedure is less than 10 hours, which is about 80% reduction in time compared to the completely patient-specific method. The major factor that allowed such a huge reduction in time is because the new approach only requires the incorporation of injury patterns and implant types to the preexisting master model, hence eliminating the need to generate new model every time the analysis is performed.

Of course, this immense time saving happens at the expense of specificity. One obvious area for improvement is to differentiate between male and female pelvises in the master model. Another limitation is that the predicted maximum stress levels in numerical simulations were lower than the actual failure stress of the implants reported in the literature. However it should be noted that the boundary conditions used in FE simulations were static conditions, which would have contributed to the overall low stress level. The use of static boundary conditions also limited the capability of our model as it cannot predict dynamic failure due to large impact.

However, our model prediction matched well with the actual clinical course qualitatively; therefore, if dynamic boundary conditions had been used, the predicted stress value would have increased, possibly to the level closer to actual failure stresses of implants.

To conclude, we presented a novel way of performing patient-specific FE analysis of pelvic ring fracture osteosynthesis. Our method uses a preexisting master model, to which patient-specific fracture patterns and implants are incorporated. This made our method a lot more efficient than completely patient-specific FE analysis that we reported previously [9]. In this novel technique presented in this paper, we have achieved considerable reductions in time and labour compared to our previous work. Although some further reductions and refinements are required, our method can be regarded as a viable first step towards developing a FE based clinical tool for surgical planning and postsurgical management of patients with pelvic ring fractures. Future works will include building a library for master models for different gender and ethnic groups as well as expanding this method to other types of fractures, implants, and bones used in this study.

## Figures and Tables

**Table 1:** List of inserted ligaments.

	Ligaments	Cross-sectional area (mm^2^)
Anterior pelvic ring	Lig. pubicum superius	7
	Lig. pubicum inferius	28
	Lig. inguinale	7
	Membrana obturatoria	10

Pelvic floor	Lig. sacrotuberale	42,85
	Lig. sacrospinale	35,6

Posterior pelvic ring	Lig. iliolumbale	21
	Lig. sacroiliacum anterior	96
	Lig. sacroiliacum posterior	17
	Lig. sacroiliacum interosseum	10

**Table 2:** Bone material properties used.

	Young's modulus [N/mm^2^]	Poisson ratio	Tensile strength (N/mm^2^)	Compression strength (N/mm^2^)
Cortical bone [14, 15]	18.000	0.3	135	205
Cancellous bone [12]	1050	0.2	7	10
Cartilage [13]	150	0.2	—	—

**Table 3:** Tension and displacement in osteosynthesis of the investigated load cases for Patient A.

Load case	Anterior osteosynthesis		Posterior osteosynthesis	
	Max. tension [MPa]	Max. displacement [mm]	Max. tension [Mpa]	Max. displacement [mm]
Getting up without support	566,86	1,53	154,81	0,18
Fast walking	70,18	2,16	64,73	0,62
Stair climbing	36,71	1,40	128,55	0,30
Stumbling	76,69	2,27	221,12	0,43

**Table 4:** Tension and displacement in the osteosynthesis of the investigated load cases for Patient B.

Load case	Anterior osteosynthesis		Posterior osteosynthesis	
	Max. tension [MPa]	Max. displacement [mm]	Max. tension [MPa]	Max. displacement [mm]
Getting up without support	24,98	1,58	82,95	0,45
Fast walking	22,57	0,89	83,34	0,58
Stair climbing	24,76	1,18	101,66	0,72
Stumbling	61,41	1,20	110,77	0,90
