# Corrigendum to “Development of a Patient-Specific Finite Element Model for Predicting Implant Failure in Pelvic Ring Fracture Fixation”

**DOI:** 10.1155/2017/8956549

**Published:** 2017-11-23

**Authors:** Vickie Shim, Andreas Gather, Andreas Höch, David Schreiber, Ronny Grunert, Steffen Peldschus, Christoph Josten, Jörg Böhme

**Affiliations:** ^1^Auckland Bioengineering Institute, University of Auckland, 70 Symonds Street, Auckland, New Zealand; ^2^Menzies Health Institute, Griffith University, Gold Coast, QLD, Australia; ^3^Department of Trauma, Plastic and Reconstructive Surgery, University of Leipzig, Liebigstr. 20, 04103 Leipzig, Germany; ^4^Institute of Forensic Medicine, Ludwig-Maximilians-University Munich, Munich, Germany

In the article titled “Development of a Patient-Specific Finite Element Model for Predicting Implant Failure in Pelvic Ring Fracture Fixation” [1], Dr. Andreas Gather, Mr. David Schreiber, and Dr. Christoph Josten were missing from the authors' list. The corrected authors' list is shown above.
